# Estimated incubation period distributions of mpox using cases from two international European festivals and outbreaks in a club in Berlin, May to June 2022

**DOI:** 10.2807/1560-7917.ES.2023.28.27.2200806

**Published:** 2023-07-06

**Authors:** Sarah E McFarland, Ulrich Marcus, Lukas Hemmers, Fuminari Miura, Jesús Iñigo Martínez, Fernando Martín Martínez, Elisa Gil Montalbán, Emilie Chazelle, Alexandra Mailles, Yassoungo Silue, Naïma Hammami, Amaryl Lecompte, Nicolas Ledent, Wim Vanden Berghe, Laurens Liesenborghs, Dorien Van den Bossche, Paul R Cleary, Jacco Wallinga, Eve P Robinson, Tone Bjordal Johansen, Antra Bormane, Tanya Melillo, Cornelia Seidl, Liza Coyer, Ronja Boberg, Annette Jurke, Dirk Werber, Alexander Bartel

**Affiliations:** 1 Unit for Surveillance and Epidemiology of Infectious Diseases, State Office for Health and Social Affairs (SOHSA), Berlin, Germany; 2 Unit 'HIV/AIDS, STI and Blood-borne Infections', Department of Infectious Disease Epidemiology, Robert Koch Institute, Berlin, Germany; 3 Postgraduate Training for Applied Epidemiology (PAE), Robert Koch Institute, Berlin, Germany; 4 ECDC Fellowship Programme, Field Epidemiology path (EPIET), European Centre for Disease Prevention and Control (ECDC), Solna, Sweden; 5 Centre for Infectious Disease Control, National Institute for Public Health and the Environment (RIVM), Bilthoven, the Netherlands; 6 Center for Marine Environmental Studies (CMES), Ehime University, Ehime, Japan; 7 Directorate General of Public Health, Regional Ministry of Health of Madrid, Madrid, Spain; 8 Santé publique France, the French national public health agency, Saint-Maurice, France; 9 Santé publique France, the French national public health agency, Paris area regional office, Saint-Denis, France; 10 Department of Infectious Disease Prevention and Control, Agency for Care and Health, Flemish Region, Brussels, Belgium; 11 Department of Epidemiology and Public Health, Sciensano, Brussels, Belgium; 12 Department of Infectious Disease Prevention and Control, Common Community Commission, Brussels-Capital Region, Brussels, Belgium; 13 Department of Clinical Sciences, Institute of Tropical Medicine, Antwerp, Belgium; 14 Field Service North West, UK Health Security Agency, Liverpool, United Kingdom; 15 Department of Biomedical Data Sciences, Leiden University Medical Center (LUMC), Leiden, the Netherlands; 16 Health Protection Surveillance Centre, Dublin, Ireland; 17 Department of Infection Control and Preparedness, Norwegian Institute of Public Health, Oslo, Norway; 18 Diseases Surveillance and Immunization Unit, Centre for Disease Prevention and Control, Riga, Latvia; 19 Infectious Disease Prevention and Control Unit, HPDP, Department for Health Regulation, Ministry of Health, Gwardamangia, Malta; 20 Bavarian Health and Food Safety Authority (LGL), Munich, Germany; 21 State Office for Occupational Safety, Consumer Protection and Health, Brandenburg, Germany; 22 NRW Centre for Health, North Rhine-Westphalia, Bochum, Germany; 23 Freie Universität Berlin, Berlin, Germany

**Keywords:** incubation period, monkeypox virus, mpox, MPXV, Germany, Europe

## Abstract

**Background:**

Since May 2022, an mpox outbreak affecting primarily men who have sex with men (MSM) has occurred in numerous non-endemic countries worldwide. As MSM frequently reported multiple sexual encounters in this outbreak, reliably determining the time of infection is difficult; consequently, estimation of the incubation period is challenging.

**Aim:**

We aimed to provide valid and precise estimates of the incubation period distribution of mpox by using cases associated with early outbreak settings where infection likely occurred.

**Methods:**

Colleagues in European countries were invited to provide information on exposure intervals and date of symptom onset for mpox cases who attended a fetish festival in Antwerp, Belgium, a gay pride festival in Gran Canaria, Spain or a particular club in Berlin, Germany, where early mpox outbreaks occurred. Cases of these outbreaks were pooled; doubly censored models using the log-normal, Weibull and Gamma distributions were fitted to estimate the incubation period distribution.

**Results:**

We included data on 122 laboratory-confirmed cases from 10 European countries. Depending on the distribution used, the median incubation period ranged between 8 and 9 days, with 5th and 95th percentiles ranging from 2 to 3 and from 20 to 23 days, respectively. The shortest interval that included 50% of incubation periods spanned 8 days (4–11 days).

**Conclusion:**

Current public health management of close contacts should consider that in approximately 5% of cases, the incubation period exceeds the commonly used monitoring period of 21 days.

**Key public health message**

**What did you want to address in this study?**

Determining time of infection for mpox among men who have sex with men is challenging as they often report multiple sexual encounters. Data from traditional investigations which determine time of infection ‘backwards’ (from outcome to exposure) can miss exposures further away from disease onset. We applied a forward-looking approach for our incubation period estimates, using data from early mpox outbreaks where infection likely occurred.

**What have we learnt from this study?**

The majority of cases had disease manifestations within 4–11 days post exposure.

Approximately 5% of cases had incubation periods of less than 3 days and 5% had incubation periods of more than 21 days.

**What are the implications of your findings for public health?**

For management of close contacts, it should be considered that some cases may not become apparent during the commonly recommended (self-)monitoring period of 21 days. Our findings that short incubation periods are possible support current recommendations that pre-exposure vaccination of high-risk groups should be considered.

## Introduction

Mpox is a viral disease endemic to Central and West Africa. It is caused by the monkeypox virus (MPXV), a member of the *Orthopoxvirus* genus which also includes smallpox [1]. Since May 2022, numerous cases have been reported, primarily in non-endemic countries worldwide [2]; from 23 July 2022 to 11 May 2023 the global outbreak was categorised as a Public Health Emergency of International Concern (PHEIC) [3]. Between 1 January and 8 December 2022, 82,522 mpox cases were reported by 110 countries across six WHO regions [4], and 25,573 cases from 45 countries and areas in the World Health Organization (WHO) European Region have been identified [5]. In the European Region, Germany had, after Spain, France and the United Kingdom (UK), the fourth highest number of reported cases [5,6].

In endemic countries, circulation of MPXV is thought to be maintained through animal reservoirs; contact with infected animals plays an important role in transmission [7]. However, human-to-human transmission as a result of close contact with lesions or body fluids, via exposure to respiratory droplets during face-to-face contact or through contact with contaminated objects (e.g. bedding) also occurs [7]. For the outbreak since May 2022 in non-endemic countries, the majority of reported cases have been due to human-to-human transmission, particularly among men who have sex with men (MSM) [2,4]. For cases with data on sexual orientation, 96% identified themselves as MSM who had been exposed through sexual contact [2].

The incubation period of mpox has historically been reported to range from 5 to 21 days and may vary depending on the type of exposure and transmission route [1,8]. Four early publications focusing on cases in the global outbreak that began in May 2022 have provided incubation period estimates based mostly on small case numbers and the most likely time of infection determined by case investigations (e.g. case interviews or questionnaires) [9-12]. Several larger studies with a main focus on clinical aspects of mpox have also described the incubation period based on exposures reported by cases [13-15]. Due to the sensitive nature of investigating cases with exposure histories involving sexual contact, uncertainty remains regarding the accuracy of self-reported exposures. The reporting of multiple potential exposures also poses a challenge with regards to the identification of transmission events and the use of case investigation data to estimate the incubation period [16]*. Adding to the knowledge base for the global outbreak using estimates from cases with a highly probable time and place of infection is therefore important as it enables more reliable incubation period estimations to inform public health recommendations, in particular for monitoring of close contacts.

We aimed to estimate the incubation period distribution of mpox by using notified cases associated with early outbreaks. To increase sample size, we invited European countries to contribute data on cases who were likely to be part of these outbreaks.

## Methods

### Case definition

We included laboratory-confirmed mpox cases notified in Europe with a reported symptom onset date and whose probable place of infection (PPOI) was a fetish festival in Antwerp, Belgium (4–9 May 2022), a gay pride festival in Gran Canaria, Spain (5–15 May 2022) or a club in Berlin, Germany (Club C, 10 May–11 June 2022). For both festivals, we included only cases who attended the festival and were exposed for 5 days or less. We assumed that exposure could have occurred shortly before or after the festivals; therefore, cases whose exposure interval overlapped with the festival dates were also included. As most cases attended the festivals for at least several days, an exposure interval cut-off of 5 days was chosen as a compromise between statistical power (i.e. sample size) and uncertainty (i.e. length of exposure interval). For Club C, an additional inclusion criterion was that cases had to be part of a cluster of five or more cases who visited Club C within a time frame of 5 days, corresponding to extended weekends in which the club was open. Cases for any of the three events were excluded if their dates (exposure and/or symptom onset) were incomplete, if they visited more than one of the events above, if their dates of exposure did not overlap with the time frame of the event(s) or if the responsible health authority considered it unlikely that the exposure was associated with infection.

### Data sources

The federal public health authority in Germany (Robert Koch Institute) contacted public health authorities from countries within the European region and invited them to send anonymised information on laboratory-confirmed mpox cases meeting the above case definition to the state health authority in Berlin, Germany (State Office for Health and Social Affairs (SOHSA)). Information requested was limited to sex, symptom onset date, first reported symptom(s), exposure date(s) and assessment of whether the reported exposure was the PPOI. The SOHSA contacted the federal states in Germany through established national networks. Countries within the European region were contacted through the European Centre for Disease Prevention and Control European Network for sexually transmitted infections (STI) surveillance, bilaterally based on established networks, or through contact information provided in recent mpox publications. We screened all submitted cases to determine if they met the inclusion criteria described above.

### Estimation and analysis of the incubation period

The probable incubation interval for each case was calculated by subtracting the start and end date of the exposure period from the date of symptom onset. To account for uncertainty regarding the exposure date, 0.5 days were subtracted from the lower limit and 0.5 days were added to the upper limit of each incubation interval. The rationale for this was that festival events and club visits often started early in the night (21:00–23:00) and lasted until the morning of the next day. In addition, the exposure for cases with a single exposure date could still have lasted for several hours, e.g. when visiting a club. Consequently, all cases were doubly censored with regards to the probable incubation period. We further assumed that the incubation period was at least 1 day and thus the lower limit of the probable incubation interval was set to 1 day, if smaller.

Since all cases had a range of probable incubation periods, we used a bootstrapping method (resamplings: n = 10,000) to provide a non-parametric estimate of the empirical distribution of the incubation period. For each case in each resampled dataset, a definite incubation period was randomly sampled from the incubation interval of the case using a uniform distribution. The resulting incubation periods were visualised using a density histogram and a smoothed density curve was calculated using a bandwidth of 2 days and a Gaussian kernel.

To estimate the parametric incubation period distribution, we fitted three doubly censored models using the log-normal, Weibull and Gamma distributions. This was done in R (3.1.4) with brms (version 2.17.0) [17] and rstan (version 2.21.2) [18]. Goodness of fit was compared using the widely applicable information criterion (WAIC), the leave-one-out information criterion (LOOIC) and the 10-fold cross-validation information criterion (R package loo version 2.5.1) [19]. The probability density function (PDF) and cumulative distribution function (CDF) were visualised for each of the three distributions based on 500 posterior samples. Credible intervals were calculated as highest density intervals. We carried out a sensitivity analysis to examine the effect of length of exposure, the influence of the case with longest incubation period and the effect of including only cases from the two festivals (fetish festival, Antwerp, Belgium and gay pride festival, Gran Canaria, Spain) and Club C in Berlin, Germany (14/15 May). Details on the sensitivity analysis are provided in Supplementary Tables S3-S7.

To explore the difference in the median incubation time between cases with skin lesions vs prodromal symptoms as the first symptoms, we used the above non-parametric method.

## Results

### Case description

Within Germany, data were provided by four German federal states. Nine other countries also provided data. In total, we had information on 222 cases: 140 from European countries (excluding Germany), 66 from the state of Berlin, the state with the highest case numbers in this outbreak [20], and 16 from three other German federal states. Overall, we included 122 cases in the analysis. Of the 100 cases excluded, 25 had incomplete exposure data, 17 had exposure dates that did not overlap with the events, seven had visited multiple events (indeterminate PPOI) and 51 had an exposure interval of more than 5 days.

The PPOI was Club C (Berlin, Germany), the gay pride festival (Gran Canaria, Spain) and the fetish festival (Antwerp, Belgium) for 58.2%, 20.5% and 21.3% of cases, respectively. Among the included cases with information on first symptom(s) (n = 69), 30 had prodromal symptoms, 33 had skin lesions and six had both prodromal symptoms and skin lesions (Table 1).

**Table 1:** Screened mpox cases included and excluded from the analysis, by country, event and first reported symptom, May–June 2022 (n = 222)

	Included			Excluded		
	n		%	n		%
Number of cases	122			100		
Median length of exposure in days (range)	1.00 (1.00–5.00)			8.00 (1.00–21.00)		
Country	122	100.0		100	100.0	
Germany^a^	71	58.2		11	11.0	
Belgium	16	13.1		21	21.0	
Spain (Madrid only)	11	9.0		16	16.0	
France	9	7.4		16	16.0	
United Kingdom	7	5.7		26	26.0	
The Netherlands	6	4.9		4	4.0	
Other^b^	2	1.6		6	6.0	
Event^c^	122	100.0		51	51.0	
Club C, Berlin	71	58.2		1	2.0	
Fetish festival, Antwerp	26	21.3		1	2.0	
Gay pride festival, Gran Canaria	25	20.5		49	96.1	
First symptom^d^	69	56.6		50	50.0	
Prodromal	30	43.5		31	62.0	
Prodromal/skin lesion	6	8.7		6	12.0	
Skin lesion	33	47.8		13	26.0	

^a^ Includes only data from the following German federal states: Bavaria, Berlin, Brandenburg, North Rhine-Westphalia.

^b^ Ireland, Latvia, Malta and Norway.

^c^ Of the 100 excluded cases, exposure for 49 could not be clearly associated with one of the three events.

^d^ Information on first symptoms was available for 119 of the 222 cases.

**Figure 1:**
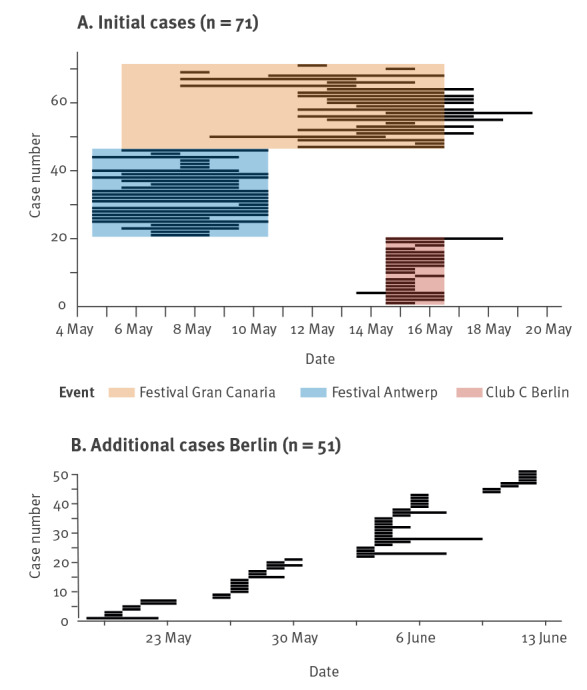
Exposure periods of included mpox cases, Berlin, May to June 2022 (n = 122)

The exposure intervals of all included cases are illustrated in Figure 1. The cases in Figure 1A were from outbreaks in the first half of May 2022 and attended the fetish festival in Antwerp, Belgium (n = 26), the gay pride festival in Gran Canaria, Spain (n = 25) or Club C in Berlin, Germany during the earliest recorded outbreak from 14 to 15 May 2022 (n = 20). Many were international cases that travelled back to their respective home countries (Antwerp, Belgium: n = 12; Gran Canaria, Spain: n = 14; Berlin, Germany: n = 4). Exposure intervals for cases involved in additional outbreaks in Club C between 18 May and 11 June 2022 (n = 51) are illustrated in Figure 1B. The majority of these cases (n = 49) were from Berlin, Germany.

Each line denotes one case. The start of the line represents the start of the exposure interval and the end of the line the end of the exposure interval. Panel A: Cases that attended the fetish festival in Antwerp, Belgium, gay pride festival in Gran Canaria, Spain or Club C in Berlin, Germany on 14–15 May 2022. Panel B: Cases from additional outbreaks related to Club C in Berlin, Germany.

### Empirical incubation periods

**Figure 2:**
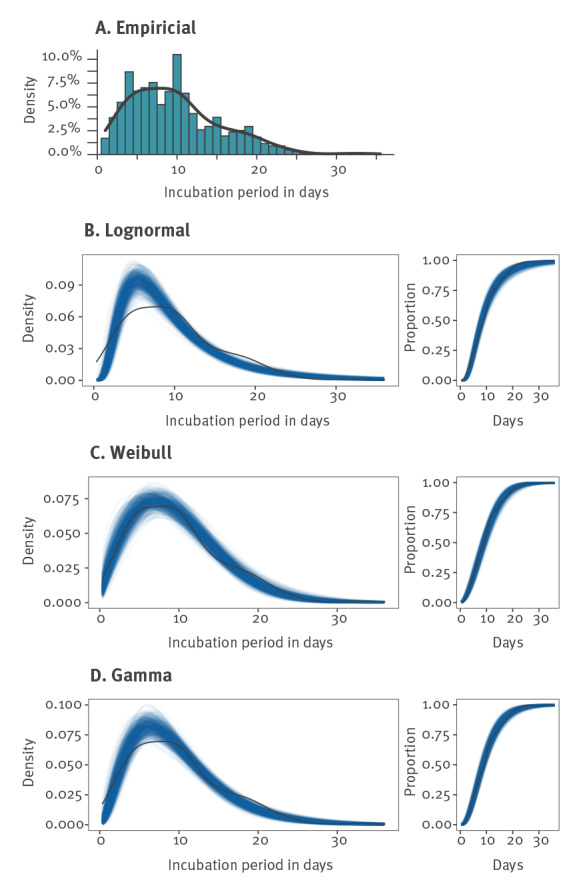
Non-parametric and parametric incubation period distributions, mpox, Berlin, May to June 2022 (n = 122)

Figure 2A shows the empirical distribution of the incubation periods. The histogram shows a plateau for the incubation period of 3–11 days, where each day had more than 5% probability of being the symptom onset. The probability of the incubation period being within this range was 64.2%. Based on the empirical density estimate, the 50% highest density interval, which is the smallest interval containing 50% of probable incubation periods, was 3.7–11.2 days.

Panel A: Empirical distribution of the incubation periods shown as a density histogram and a smoothed density curve. Panels B–C left graph: Posterior samples of the probability density function of the fitted log-normal, Weibull and gamma distributions. The black line denotes the empirical smoothed density curve. Panels B–C right graph: Posterior samples of the cumulative distribution function of the fitted log-normal, Weibull and gamma distributions. The black line denotes the empirical cumulative distribution.

Five per cent of the probable values accounted for an incubation period of 2 days or less. Six cases had a probable incubation period above 21 days. The upper limit of the probable incubation period of five of these cases was shorter than 26 days, whereas one case had a probable incubation period of 30–35 days. We observed a spike in the empirical density for an incubation period of 10 days. Further analysis found only a small possible bias caused by the reported weekday for the nine cases that had an incubation period of exactly 10 days. Only three of the nine cases were exposed on Friday and reported symptoms on Monday 10 days later. This was only marginally more than the one to two randomly expected cases for nine cases spread over seven weekdays.

The percentiles and the medians of the incubation period were similar across the different distributions: 2.1 to 2.9 days for the 5th percentile and 8.1 to 9.0 days for the median (Figure 2B, Table 2). For the 95% percentile, the uncertainty was higher. The Weibull and Gamma distribution estimates were 20.1 and 20.6 days, respectively, whereas the log-normal distribution estimate was 23.1 days.

**Table 2:** Empirical and estimated percentiles of the incubation period in days for mpox based on different distributions, Berlin, May to June 2022 (n = 122)

Quantile	Empirical	Lognormal	Weibull	Gamma
2.5%	1.7	2.4 (1.9–2.8)	1.5 (1.0–2.0)	1.9 (1.3–2.5)
5%	2.3	2.9 (2.3–3.4)	2.1 (1.6–2.8)	2.5 (1.9–3.2)
10%	3.3	3.6 (3.0–4.2)	3.2 (2.4–3.9)	3.5 (2.8–4.2)
25%	5.3	5.3 (4.6–6.0)	5.5 (4.7–6.5)	5.5 (4.7–6.3)
50%	9.0	8.1 (7.2–9.1)	9.0 (7.9–10.0)	8.6 (7.7–9.6)
75%	12.8	12.5 (11.0–14.1)	13.1 (11.9–14.5)	12.7 (11.4–14.1)
90%	18.3	18.3 (15.7–21.3)	17.4 (15.6–19.3)	17.4 (15.4–19.5)
95%	20.4	23.1 (19.3–27.5)	20.1 (18.0–22.5)	20.6 (18.2–23.4)
97.5%	22.7	28.1 (23.0–34.3)	22.5 (19.9–25.4)	23.7 (20.7–27.1)
99%	25.5	35.5 (28.3– 44.4)	25.4 (22.2–29.0)	27.6 (23.9–31.9)

Estimates are shown with 95% credible intervals in parentheses.

The three models had similar predictive performances; the difference in goodness of fit was small (i.e. below 4) for all information criteria (IC) [21,22]. The WAIC showed a maximal difference of 2.8, LOOIC of 2.8 and the 10-fold cross-validation information criterion (10-fold IC) of maximal 1.5. In Supplementary Table S2, we provide the values of the WAIC, LOOIC and 10-fold IC for each distribution. The log-normal distribution estimated a lower probability of shorter incubation periods and a higher probability of longer incubation periods. In particular for the CDF, the log-normal distribution lay below the empirical cumulative density function around day 23. In Supplementary Table S1 we provide the estimated distribution parameters. 

Explorative analysis of cases with skin lesions vs prodromal symptoms as the first reported symptom showed that the median empirical incubation period of 9.8 days for cases with skin lesions (n = 33) was longer than the median empirical incubation period of 9.0 days for cases with prodromal symptoms (n = 30).

Because we estimated upper 95% limits of the incubation period distribution of more than 20 days, we investigated the influence of the case with the longest reported incubation period interval (30–35 days). Exclusion of this case resulted in a shortening of the 95% quantile by 0.6 to 0.9 days, which was still well within the original credible intervals. Changes in the median and the 5% quantiles were within the rounding error (0.2 days) (see Supplementary Table S3 for the full results). The length of the exposure interval had little effect on the incubation period estimation as the credible intervals had considerable overlap (> 70%) and their width did not vary. Sensitivity analysis results on the length of exposure can be accessed in Supplementary Tables S6-S8.

## Discussion

We estimated the incubation period distribution for mpox using cases for whom the time (interval) of infection could be determined with reasonable confidence. All three distributions used fitted the data well. Our study corroborates the wide range of possible mpox incubation periods, which can be as short as 2–3 days (5% percentile of the incubation period distributions) but also longer than 20 days (95% percentile of 20, 21 and 23 days). Half of the cases had incubation periods between 4 and 11 days; the median incubation period for all cases was between 8 and 9 days.

Our estimate of the 95% percentile indicated that incubation periods longer than 21 days occurred in ca 5% of cases. This is comparable to results from previous studies, which estimated 95th percentiles of 17, 18 and 20 days [9-12]. The two studies that estimated a 95th percentile of 17 days were small initial studies from June 2022 that partly shared cases [9,10]. Because these studies aimed to provide early actionable information on the incubation period, they had a short follow-up time. In addition, some cases with longer incubation periods may not have been captured as in a growing outbreak, infected persons with a long incubation period have a lower probability of being included (right-censoring bias) [23]. Furthermore, in routine case investigations by public health personnel, incubation periods are estimated ‘backwards’ from disease onset to possible exposures. This approach, which has also been used in previous studies on the mpox incubation period [9-12], is subject to backward biases, especially when incidence is increasing [23]. In addition, under-ascertainment of exposures that have occurred near or outside presumed maximal incubation periods (e.g. 21 days for mpox) can occur, particularly when plausible exposures were closer to disease onset. To account for this, we conceptually estimated the incubation period ‘forward’, analogous to a retrospective cohort study, starting from the likely time point of infection [23]. We also included only cases from early outbreaks in our study as it was likely that infection occurred in these settings, particularly because MPXV was not widespread at that time and risk of infection outside of these outbreaks was therefore comparatively low. With this approach, it is less likely that long incubation periods were due to unobserved (secondary) exposures that occurred after the presumed time of infection [24]. In addition, as the follow-up time for included cases in our study was at least 45 days, our study was less susceptible to underrepresentation of cases with longer incubation periods.

The 5% percentiles of 2 and 4 days from two studies [9,11] are in line with our estimate of 2–3 days and support our findings that very short incubation periods are possible. This, along with findings of possible presymptomatic transmission of mpox [12,25], has implications for vaccination strategies. In particular, the effectiveness of post-exposure vaccination strategies may be limited and pre-exposure vaccination of high-risk groups should be emphasised, provided that a sufficient number of vaccine doses are available [13,25,26].

The comparatively larger number of cases included in our study enabled more precise estimates of the incubation period than previous studies. This is illustrated by the narrow credible intervals for our distribution estimates, with only ± 1 day uncertainty for the median estimates. In addition, sensitivity analysis showed that removing the case with the highest incubation period had little effect on the incubation period estimates. This underscores that the estimates are robust against single outlier observations.

To evaluate if estimates depend on the distribution(s), different distributions should be used to compare incubation period estimates [27]. Based on our results, the chosen distribution had little effect on the incubation period estimates as all had a similar goodness of fit. The largest difference was observed in the 95% percentile, with 20.1 and 20.6 days for the Weibull and Gamma distributions and 23.1 days for the log-normal distribution due to its long tail. The right tail of the log-normal distribution deviated substantially from the empirical data and estimated the highest possible 95th percentile, representing the most conservative scenario.

Incubation period estimates for cases who had skin lesions as their first symptom vs cases that had prodromal symptoms showed less than a day difference. Our observed median incubation time of 9.8 days for cases with skin lesions as the first symptom was longer than the estimate of 7.8 days in a previous study [10]. Several recent large studies with a broad focus on clinical characteristics of mpox have also reported incubation period results. As they have time frames and methods that are not tailored to only assess the incubation period of mpox, comparing our results with these studies is difficult [13-15].

Our study is subject to limitations. Initial symptoms (e.g. fatigue, fever) can be non-specific; therefore, it is possible that they were not related to mpox illness or that early symptoms were overlooked, potentially biasing our estimates. Notably, published clinical findings for this global mpox outbreak have described previously unreported clinical presentations including single lesions in or around the genitals and anus, oral lesions and symptoms of proctitis [13,15]. This type of clinical presentation might initially have been missed by cases as well as physicians and may have resulted in a slight overestimation of the incubation period in our study and in previous studies, particularly, as we included cases early in the mpox outbreak when knowledge was limited. Furthermore, incomplete recall could have affected our estimates, especially since multiple sexual contacts were commonly reported. Therefore, we cannot completely exclude that the cases, although present at a festival or club with recognised outbreaks, were infected at a later time. Lastly, data regarding smallpox vaccination status was not available for the majority of cases. As a result, we were unable to evaluate the possible influence of vaccination status on incubation period.

## Conclusion

Information on the incubation periods for mpox in the global outbreak beginning in May 2022 is important to inform public health recommendations aimed at limiting onward transmission, such as the time period for self-monitoring of contact persons or restrictions for close contacts. Our study illustrates the benefit of collaborative collection and analysis of cases in novel outbreaks in order to build a knowledge base to guide public health recommendation and measures. It also includes the largest number of cases to date used in published studies modelling incubation period estimates for the 2022 global outbreak. Approximately 5% of cases have an incubation period greater than 21 days, and therefore may not become apparent during the commonly recommended monitoring period of close mpox contacts.

## *Erratum

In the version originally published on 6 July, the references from number [16] onwards were misnumbered. The reference numbering was corrected on 10 July 2023. We apologise for any inconvenience this may have caused.

## Supplementary Data

198000 Supplement

## Supplementary Data

8000 Supplement Data

## Supplementary Data

14000 Supplement Code
