# Erratum for Euro Surveill. 2023;28(27)

**DOI:** 10.2807/1560-7917.ES.2023.28.28.230713e

**Published:** 2023-07-13

**Authors:** Eurosurveillance editorial team

**Affiliations:** 1 European Centre for Disease Prevention and Control (ECDC), Stockholm, Sweden

In the article entitled ‘Estimated incubation period distributions of mpox using cases from two international European festivals and outbreaks in a club in Berlin, May to June 2022’ by McFarland et al., published on 6 July 2023, the references from number [16] onwards were misnumbered. The reference numbering was corrected on 10 July 2023. We apologise for any inconvenience this may have caused.

