# Supplementary material for: Estimated incubation period distributions of mpox using cases from two international European festivals and outbreaks in a club in Berlin, May to June 2022
Source: Euro Surveill. 2023 Jul 6;28(27):2200806. doi: 10.2807/1560-7917.ES.2023.28.27.2200806 (PMC10370040; doi:10.2807/1560-7917.ES.2023.28.27.2200806)
Supplement: Supplement [file 22-00806_BARTEL_Supplement.pdf]

Eurosurveillance

Supplement for “Estimated incubation period distributions of monkeypox using cases from two international European festivals and outbreaks in a club in Berlin, May – June 2022”

"This supplementary material is hosted by *Eurosurveillance* as supporting information alongside the article “Estimated incubation period distributions of monkeypox using cases from two international European festivals and outbreaks in a club in Berlin, May – June 2022”, on behalf of the authors, who remain responsible for the accuracy and appropriateness of the content. The same standards for ethics, copyright, attributions and permissions as for the article apply. Supplements are not edited by *Eurosurveillance* and the journal is not responsible for the maintenance of any links or email addresses provided therein."

Table S1: Parameters of the incubation period distributions (posterior median + 95% credible interval)

Weibull:

- Intercept: 2.28 (2.17– 2.38)
- Shape: 1.81 (1.57 – 2.08)
- Scale: 10.98 (9.84 – 12.11)

Lognormal:

- Intercept: 2.10 (1.98– 2.21)
- SD: 0.63 (0.55 – 0.72)

Gamma:

- Intercept: 0.10 (0.09 – 0.11)
- Shape: 2.87 (2.19 – 3.64)
- Rate: 0.30 (0.22 – 0.38)
- Scale: 3.37 (2.53 – 4.38)

Goodness of fit

Table S2. Computed goodness of fit of the models based on three parametric distributions using the widely applicable information criterion (WAIC), the leave-one-out information criterion (LOOIC) and the 10-fold cross-validation information criterion (10-fold IC).

Distribution	WAIC*	LOOIC*	10-fold IC*
**Lognormal**	-297.2	-297.2	-296.9
**Weibull**	-295.7	-295.7	-295.4
**Gamma**	-294.4	-294.4	-296.1

*Goodness of fit estimates, higher values indicate a better fit.

Sensitivity Analysis

1. Table S3: All cases excluding one outlier case with an incubation period of 30 to 35 days.

n = 121

Quantile	Lognormal	Weibull	Gamma
**2,5%**	2.4 (1.9 - 2.9)	1.6 (1.1 - 2.1)	2 (1.4 - 2.5)
**5%**	2.9 (2.4 - 3.4)	2.3 (1.7 - 2.9)	2.6 (2 - 3.2)
**10%**	3.6 (3 - 4.2)	3.3 (2.6 - 4)	3.5 (2.8 - 4.2)
**25%**	5.3 (4.6 - 6)	5.6 (4.7 - 6.5)	5.5 (4.7 - 6.3)
**50%**	8 (7.2 - 9)	8.9 (7.9 - 9.9)	8.5 (7.6 - 9.5)
**75%**	12.2 (10.8 - 13.8)	12.8 (11.6 - 14)	12.4 (11.1 - 13.8)
**90%**	17.8 (15.3 - 20.8)	16.7 (15.1 - 18.5)	16.9 (15 - 19)
**95%**	22.4 (18.7 - 26.7)	19.2 (17.2 - 21.4)	19.9 (17.6 - 22.7)
**97,5%**	27.2 (22.2 - 33)	21.4 (19.1 - 24.1)	22.9 (20.1 - 26.3)
**99%**	34.2 (27.2 - 42.6)	24.1 (21.2 - 27.4)	26.6 (23.1 - 30.8)

2. Table S4: All cases that attended the fetish festival (Antwerp, Belgium), gay pride festival (Gran Canaria, Spain) and Club C (Berlin, Germany; 14/15 May 2022).

n = 71

Quantile	Lognormal	Weibull	Gamma
**2,5%**	3 (2.1 - 3.8)	2 (1.2 - 2.8)	2.5 (1.5 - 3.4)
**5%**	3.6 (2.7 - 4.5)	2.9 (1.9 - 3.9)	3.2 (2.2 - 4.2)
**10%**	4.5 (3.6 - 5.5)	4.2 (3 - 5.4)	4.3 (3.2 - 5.5)
**25%**	6.5 (5.4 - 7.6)	6.9 (5.5 - 8.3)	6.7 (5.5 - 8)
**50%**	9.8 (8.4 - 11.3)	10.8 (9.4 - 12.4)	10.3 (8.9 - 11.8)
**75%**	14.8 (12.6 - 17.4)	15.4 (13.6 - 17.4)	15 (13.1 - 17.2)
**90%**	21.5 (17.6 - 26.2)	20 (17.6 - 22.8)	20.2 (17.4 - 23.6)
**95%**	26.8 (21.4 - 33.7)	22.8 (19.8 - 26.2)	23.9 (20.3 - 28.1)
**97,5%**	32.5 (25.3 - 42.2)	25.4 (21.8 - 29.5)	27.3 (23 - 32.6)
**99%**	40.6 (30.3 - 54.4)	28.4 (24.3 - 33.6)	31.8 (26.3 - 38.4)

3. Table S5: All cases that attended the fetish festival (Antwerp, Belgium), gay pride festival (Gran Canaria, Spain) and Club C (Berlin, Germany; 14/15 May 2022) independent of the length of exposure.

n =120

Quantile	Lognormal	Weibull	Gamma
**2,5%**	2.2 (1.7 - 2.8)	1.5 (1 - 2.1)	1.8 (1.2 - 2.5)
**5%**	2.8 (2.2 - 3.4)	2.2 (1.5 - 2.9)	2.5 (1.7 - 3.2)
**10%**	3.5 (2.8 - 4.3)	3.3 (2.5 - 4.2)	3.5 (2.6 - 4.3)
**25%**	5.4 (4.5 - 6.2)	5.9 (4.8 - 6.9)	5.7 (4.7 - 6.7)
**50%**	8.6 (7.5 - 9.8)	9.7 (8.4 - 10.9)	9.2 (8.1 - 10.4)
**75%**	13.7 (11.9 - 15.8)	14.4 (12.9 - 15.9)	13.9 (12.4 - 15.6)
**90%**	20.8 (17.5 - 24.8)	19.1 (17.2 - 21.5)	19.4 (17 - 21.9)
**95%**	26.8 (21.9 - 32.8)	22.2 (19.8 - 25.1)	23.2 (20.2 - 26.5)
**97,5%**	33.3 (26.7 - 41.9)	25 (21.9 - 28.5)	26.9 (23.1 - 31)
**99%**	42.9 (33.1 - 55.4)	28.4 (24.6 - 32.8)	31.5 (26.9 - 36.7)

4. Effect of the length of exposure (in days) on the incubation period estimation.

Table S6: Lognormal

Quantile/length in days	4 (n=104)	5 (n=122)	6 (n=130)	8 (n=146)	10 (n=160)
2,5%	2.3 (1.8 - 2.8)	2.4 (1.9 – 2.8)	2.4 (1.9 - 2.9)	2.1 (1.7 - 2.5)	2.1 (1.7 - 2.5)
5%	2.8 (2.2 - 3.3)	2.9 (2.3 – 3.4)	2.9 (2.4 - 3.4)	2.6 (2.1 - 3.1)	2.6 (2.2 - 3.1)
10%	3.5 (2.9 - 4.1)	3.6 (3.0 – 4.2)	3.6 (3.1 - 4.2)	3.3 (2.8 - 3.8)	3.3 (2.8 - 3.9)
25%	5.1 (4.3 - 5.8)	5.3 (4.6 – 6.0)	5.3 (4.7 - 6)	5 (4.4 - 5.6)	5 (4.4 - 5.7)
50%	7.7 (6.8 - 8.7)	8.1 (7.2 – 9.1)	8.1 (7.2 - 9)	7.9 (7 - 8.8)	7.9 (7.1 - 8.8)
75%	11.7 (10.2 - 13.4)	12.5 (11.0 – 14.1)	12.3 (10.9 - 13.9)	12.4 (11 - 14.1)	12.4 (11 - 14)
90%	17.1 (14.5 - 20.3)	18.3 (15.7 – 21.3)	17.9 (15.5 - 20.9)	18.7 (16.1 - 21.8)	18.7 (16.2 - 21.6)
95%	21.5 (17.7 - 25.9)	23.1 (19.3 – 27.5)	22.4 (18.9 - 26.6)	23.9 (20.2 - 28.6)	23.9 (20.3 - 28.3)
97,5%	26.2 (21.2 - 32.5)	28.1 (23.0 – 34.3)	27.3 (22.5 - 33.1)	29.6 (24.4 - 36)	29.5 (24.2 - 35.4)
99%	32.9 (25.9 - 41.9)	35.5 (28.3 – 44.4)	34.3 (27.5 - 42.6)	37.9 (30.2 - 47.2)	37.7 (30.2 - 46.3)

Table S7: Weibull

Quantile/length in days	4 (n=104)	5 (n=122)	6 (n=130)	8 (n=146)	10 (n=160)
2,5%	1.4 (0.9 - 1.9)	1.5 (1.0 – 2.0)	1.5 (1 - 2)	1.3 (0.9 - 1.8)	1.3 (0.9 - 1.8)
5%	2.1 (1.5 - 2.7)	2.1 (1.6 – 2.8)	2.1 (1.6 - 2.7)	2 (1.5 - 2.5)	2 (1.5 - 2.5)
10%	3.1 (2.3 - 3.8)	3.2 (2.4 – 3.9)	3.2 (2.5 - 3.9)	3 (2.3 - 3.7)	3 (2.4 - 3.7)
25%	5.3 (4.4 - 6.2)	5.5 (4.7 – 6.5)	5.5 (4.6 - 6.4)	5.3 (4.5 - 6.2)	5.3 (4.6 - 6.2)
50%	8.5 (7.4 - 9.5)	9.0 (7.9 – 10.0)	8.9 (7.9 - 9.9)	8.8 (7.8 - 9.8)	8.8 (7.9 - 9.8)
75%	12.3 (11.1 - 13.7)	13.1 (11.9 – 14.5)	13 (11.8 - 14.3)	13 (11.8 - 14.3)	13.1 (12 - 14.4)
90%	16.2 (14.5 - 18.1)	17.4 (15.6 – 19.3)	17.1 (15.5 - 18.9)	17.4 (15.8 - 19.3)	17.5 (15.9 - 19.3)
95%	18.7 (16.7 - 21.2)	20.1 (18.0 – 22.5)	19.7 (17.7 - 22.1)	20.2 (18.1 - 22.5)	20.3 (18.4 - 22.6)
97,5%	20.9 (18.5 - 23.9)	22.5 (19.9 – 25.4)	22.1 (19.7 - 24.9)	22.8 (20.3 - 25.6)	22.9 (20.5 - 25.7)
99%	23.6 (20.5 - 27.2)	25.4 (22.2 – 29.0)	25 (22 - 28.4)	25.9 (22.9 - 29.4)	26 (23.1 - 29.4)

Table S8: Gamma

Quantile/length in days	4 (n=104)	5 (n=122)	6 (n=130)	8 (n=146)	10 (n=160)
**2,5%**	1.9 (1.3 - 2.4)	1.9 (1.3 – 2.5)	1.9 (1.4 - 2.5)	1.7 (1.2 - 2.2)	1.7 (1.3 - 2.2)
**5%**	2.5 (1.8 - 3.1)	2.5 (1.9 – 3.2)	2.6 (2 - 3.2)	2.3 (1.7 - 2.9)	2.3 (1.8 - 2.9)
**10%**	3.3 (2.6 - 4)	3.5 (2.8 – 4.2)	3.5 (2.8 - 4.2)	3.2 (2.6 - 3.9)	3.2 (2.6 - 3.9)
**25%**	5.2 (4.4 - 6)	5.5 (4.7 – 6.3)	5.5 (4.7 - 6.3)	5.2 (4.5 - 6)	5.3 (4.6 - 6)
**50%**	8.1 (7.2 - 9.1)	8.6 (7.7 – 9.6)	8.5 (7.6 - 9.4)	8.4 (7.5 - 9.3)	8.4 (7.6 - 9.3)
**75%**	11.9 (10.6 - 13.4)	12.7 (11.4 – 14.1)	12.5 (11.3 - 13.9)	12.6 (11.4 - 14)	12.7 (11.5 - 14)
**90%**	16.2 (14.3 - 18.5)	17.4 (15.4 – 19.5)	17 (15.2 - 19.1)	17.5 (15.7 - 19.6)	17.6 (15.7 - 19.6)
**95%**	19.2 (16.8 - 22)	20.6 (18.2 – 23.4)	20.2 (17.8 - 22.8)	20.9 (18.5 - 23.6)	21 (18.7 - 23.6)
**97,5%**	22.1 (19 - 25.4)	23.7 (20.7 – 27.1)	23.2 (20.3 - 26.4)	24.1 (21.2 - 27.5)	24.2 (21.4 - 27.4)
**99%**	25.7 (22 - 30)	27.6 (23.9 – 31.9)	27 (23.4 - 31)	28.3 (24.6 - 32.5)	28.4 (24.9 - 32.5)
